# IMPROVING QUALITY IN ASSISTED LIVING: FINDINGS FROM STATEWIDE INITIATIVES

**DOI:** 10.1093/geroni/igae098.0501

**Published:** 2024-12-31

**Authors:** Sheryl Zimmerman, Philip Sloane

**Affiliations:** Chair; Discussant

## Abstract

Almost 1.7 million people in the U.S. receive residential long-term care, more than half who reside in assisted living -- broadly defined as congregate settings that provide at least two meals a day, around-the-clock supervision, and help with personal care. Whereas nursing homes are federally regulated, assisted living is state regulated, which recognizes it as a community-based setting and allows for innovative practices and policies to promote better care and outcomes. Over the years, numerous state-based initiatives have been enacted across the country; this session will discuss new initiatives in two states, addressing their implementation, findings, and broad implications. Minnesota’s legislation recently created an online report card based on resident and family surveys; the presentation by Dr. Shippee will overview three years of data from more than 12,000 residents, noting the areas scored most favorably (e.g., environment) and least favorably (e.g., engagement). Dr. Moone will discuss the Minnesota assisted living regulatory framework and how the absence of broad representation in licensure development resulted in inconsistency in and preparation for survey inspections, as well as inadequate reimbursement. North Carolina recently undertook a statewide randomized trial of accreditation for assisted living; Ms. Efird-Green will discuss challenges implementing accreditation in more than half of the communities, and Dr. Zimmerman will present two years of outcome data comparing control and accreditation arms in relation to workforce and resident outcomes, care coordination and transitions, medication management, and person-centered care. Results will be put in context to inform quality initiatives across the country.

